# Graphene Templated DNA Arrays and Biotin-Streptavidin Sensitive Bio-Transistors Patterned by Dynamic Self-Assembly of Polymeric Films Confined within a Roll-on-Plate Geometry

**DOI:** 10.3390/nano10081468

**Published:** 2020-07-27

**Authors:** Sangheon Jeon, Jihye Lee, Rowoon Park, Jeonghwa Jeong, Min Chan Shin, Seong Un Eom, Jinyoung Park, Suck Won Hong

**Affiliations:** 1 Department of Cogno-Mechatronics Engineering, Department of Optics and Mechatronics Engineering, College of Nanoscience and Nanotechnology, Pusan National University, Busan 46241, Korea; sangheon.jn@gmail.com (S.J.); jihyelee970@gmail.com (J.L.); rowoon.p153@gmail.com (R.P.); 2jeong.s.o@gmail.com (J.J.); mch3024@gmail.com (M.C.S.); 2 Substrate & Material Laboratory, LG Innotek Co., Ltd., Gumi 39419, Korea; sueom89@gmail.com; 3 School of Applied Chemical Engineering, Kyungpook National University, Daegu 41566, Korea

**Keywords:** self-assembly, graphene, DNA, biosensor, field-effect transistor

## Abstract

Patterning of surfaces with a simple strategy provides insights into the functional interfaces by suitable modification of the surface by novel techniques. Especially, highly ordered structural topographies and chemical features from the wide range of interfaces have been considered as important characteristics to understand the complex relationship between the surface chemistries and biological systems. Here, we report a simple fabrication method to create patterned surfaces over large areas using evaporative self-assembly that is designed to produce a sacrificial template and lithographic etch masks of polymeric stripe patterns, ranging from micrometer to nanoscale. By facilitating a roll-on-plate geometry, the periodically patterned surface structures formed by repetitive slip-stick motions were thoroughly examined to be used for the deposition of the Au nanoparticles decorated graphene oxide (i.e., AuNPs, ~21 nm) and the formation of conductive graphene channels. The fluorescently labeled thiol-modified DNA was applied on the patterned arrays of graphene oxide (GO)/AuNPs, and biotin-streptavidin sensitive devices built with graphene-based transistors (GFETs, effective mobility of ~320 cm^2^ V^−1^ s^−1^) were demonstrated as examples of the platform for the next-generation biosensors with the high sensing response up to ~1 nM of target analyte (i.e., streptavidin). Our strategy suggests that the stripe patterned arrays of polymer films as sacrificial templates can be a simple route to creating highly sensitive biointerfaces and highlighting the development of new chemically patterned surfaces composed of graphene-based nanomaterials.

## 1. Introduction

Over the past decades, there has been considerable progress in the development of multifunctional electronic devices capable of detecting chemical and biological substances and measuring nanoscale phenomena [1]. Recent advances in integrated circuit technology and controlled nanofabrication with nanomaterials have enabled implementations of microelectronic sensors with a variety of novel sensing mechanisms in conjunction with their collective properties [2], which can provide viable prototype solutions in applications ranging from environmental monitoring to biomedical diagnostic sensors [3]. Notably, many engineering aspects of nanoscale materials including semiconducting nanowires [4], carbon nanomaterials [5], nanoparticles [6], and conductive polymers [7] also offer new opportunities to change the face of nanoprobes or nanosensors overcoming existing challenges in bioelectronics owing to the high surface-to-volume ratios and superb physicochemical properties such as conductivity, permeability, and chemical reactivity [8]. Such integrated devices consisted of promising nanomaterials that have been proven to be highly sensitive for detecting various types of chemical or biological molecules with tunable electron transport properties owing to the quantum confinement effect [9]. Therefore, specific nanostructured materials can be used for the efficient electron transport channels in one or more dimensions [10], as well as for the enhanced biointerfaces comparable to the characteristic features of small molecules such as DNA [11]. Both of these factors make them critical to the integration of electronics devices and functions, thus rationally combining nanoelectronics and biological systems to expand the interdisciplinary strategy towards a wide range of applications [12].

In this context, among other nanomaterials, graphene, two-dimensional (2D) single-layer one-atom-thick graphitic carbon material has attracted widespread interest owing to its exceptional electronic, optical, chemical, thermal, and mechanical properties [13]. Hence, tremendous efforts have been made in the research field of graphene and graphene derivatives to utilize its extraordinary properties for technological applications such as flexible electronics [14], energy storage [15], optoelectronics [16], photonics [17], bioelectronics [18], bionic implant [19], and other graphene-based devices [20]. In particular, the electrical detection of small molecules using graphene-based devices has emerged as a strong alternative for the next-generation sensory platform owing to its ultrathin nature and tunable surface chemistry with ease of manipulation [21]. As demonstrated earlier, graphene-based nanomaterials are biocompatible and surface-responsive in the physical and chemical environment [22], which can thereby make them well suited for future biosensor platforms [23]. To date, the biosensor platforms have been developed with a variety of techniques, such as electrochemical detection using a standard glassy carbon electrode, inkjet-printed or screen-printed carbon electrodes, and optoelectronic analysis [24]. Recently, much effort has been concentrated on the fabrication of graphene field-effect transistors (GFETs), employing the patterned graphene nanosheets as conducting layers in advanced types of electrical sensor systems in combination with easily compatible fabrication processes [25]. The potential of these graphene devices has been demonstrated by several sophisticated approaches capable of detecting various types of small biomolecules and monitoring cellular responses. For these applications, the formation of the parallel aligned arrays of graphene film is indispensable to characterize test structures with a controllable configuration in a scalable and cost-effective manner, which leads to quantification of the chemical and electrical properties in the specific device structures. Therefore, it is of key importance to provide alternative patterning methods to achieve microchannels with graphene-based nanomaterials without the damage or degradation of its chemical integrity and electronic transport property [26].

Herein, we report a facile approach to fabricate highly aligned stripe mask patterns with lines and spacing configuration, using a simple upper moving roll on a flat substrate to confine the evaporative droplet of a polymer solution. After the drying of the polymer solution in the dynamic self-assembly, a so-called “coffee ring” pattern can be formed in gradient stripes [27]. The effects of the solution concentration and the moving speed of the roll on a flat surface were thoroughly examined. These patterned polymer films were readily used for the sacrificial templates or lithographic etch masks. The nanometer height of the polymeric template was fully utilized by the simple deposition of colloidal graphene oxide (GO)/AuNPs, and then a thiolate functionalized DNA solution was applied to the patterned GO/AuNPs substrate for the selective immobilization. In addition, the self-assembled polymeric stripes on the chemical vapor deposition (CVD) grown monolayer graphene sheet to build aligned arrays of graphene channels for the biomolecule adsorption in the monolithically integrated GFET-structure to detect biotin/streptavidin recombination. As a result, careful analysis of the extracted data from the devices confirmed the selective detection of a specific protein. The chemically tuned recognition layer on the graphene surface used in this study is crucial for biomolecule detection, which prevents the nonspecific binding usually observed in the ligand–receptor complex systems [28]. Our presented strategy may suggest a simple route to incorporating 2D graphene nanomaterials and the change of device characteristics by employing chemical reactions into the label-free detection of protein binding events [29].

## 2. Materials and Methods

### 2.1. Roll-on-Plate Confined Geometry

**Figure 1:**
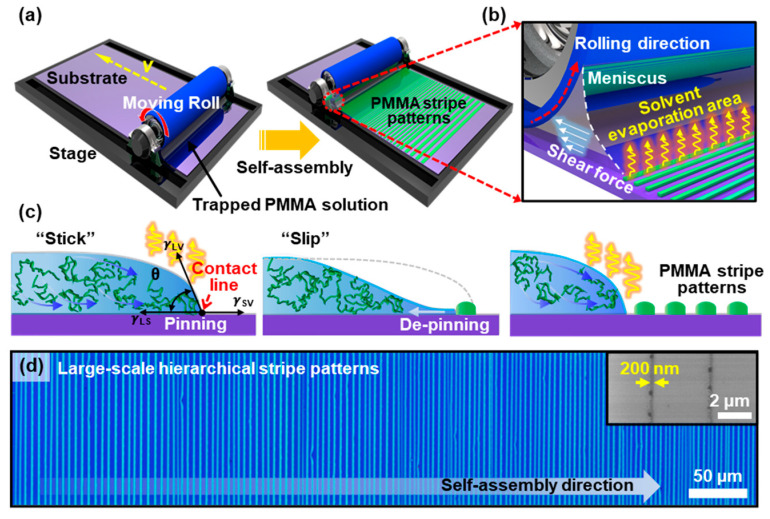
(**a**) Schematic illustration of the roll-on-plate geometry to form gradient stripe patterns by the solvent evaporation and controlled flow of PMMA solution. (**b**) Solvent evaporation of receding meniscus, dragged by a shear force, in confined geometry. (**c**) A cross-sectional view of cyclic stick-slip motions at the edge of the contact line, balanced with the capillary force and surface tension on a flat substrate. (**d**) Representative optical micrograph of highly ordered PMMA hierarchical stripes formed over a large area, perpendicular to the direction of the moving meniscus. Patterned stripes of PMMA in nanoscale at the final stage of the process (inset).

To produce the highly-ordered arrays of poly (methyl methacrylate) (PMMA) stripes, Si substrate and cylindrical tube (i.e., roll) made from fused silica (the radius of curvature, *R* = 1.33 cm, and the diameter, *D* = 1.5 cm) were used as a upper moving surface and flat substrate, respectively, constructing a unique confined geometry to trap a polymer solution, as schematically illustrated in Figure 1a. PMMA (Mw = 401 K, PDI = 1.3, Polymer Source, Quebec, Canada), was used as the nonvolatile solute to prepare polymer solutions in toluene with selected concentrations (i.e., *C* = 0.5, 0.25, and 0.125 mg mL^−1^). The dissolved PMMA solutions were filtered with a 0.2 μm hydrophobic membrane filter. Prior to use, Si substrate and cylindrical roll were cleaned with piranha solution (sulfuric acid: hydrogen peroxide = volume ratio of 7:3) imposing hydrophilicity on the surfaces, and then extensively rinsed with deionized water and dried by blowing N_2_ gas. The moving roll was controlled by the linear motorized translation stage fixed with both ends, and the cylindrical surface was in full contact with the lower stationary flat substrate (i.e., SiO_2_/Si or graphene/SiO_2_/Si). Prior to the close-contact between the roll and the flat substrate, 30 μL of PMMA solution was loaded and trapped following the roll and flat surface owing to capillary forces. Finally, the upper roll was traveled at an optimized speed of 7 μm s^−1^, leading to the spontaneous formation of the PMMA stripe patterns toward the opposite direction. These fabrication procedures modulated the dimensions of PMMA stripes with only the solution concentration (i.e., 0.5, 0.25, and 0.125 mg mL^−1^) during the evaporation process controlled by a constant roll speed at ambient conditions (~24 °C).

### 2.2. Synthesis of the Au Nanoparticles Decorated Graphene Oxide (GO/AuNPs)

The exfoliated GO solution and the GO/AuNPs composite were synthesized by our previously reported method via the modified hydrothermal method [30]. In a typical procedure, 5 mL GO aqueous solution (2 mg mL^−1^), 0.250 mL of an aqueous 0.01 M solution of gold chloride trihydrate (HAuCl_4_·3H_2_O, Sigma Aldrich, St. Louis, MO, USA) was added to 7.5 mL of a 0.10 M Hexadecyl-trimethylammonium bromide (CTAB, Sigma Aldrich, St. Louis, MO, USA) solution. Subsequently, the mixed solution was processed by ultrasonication for 8 h at room temperature, and then 10 mL of the mixture was transferred to a Teflon-lined autoclave for the hydrothermal reaction at 180 °C for 12 h. The as-prepared hybrids were cooled naturally to room temperature.

### 2.3. Fabrication of GO/AuNPs Patterns and DNA Immobilization

The GO/AuNPs solution was uniformly deposited on the gradient PMMA stripe patterns via the flow-enabled self-assembly (FESA) process, as described in our previous report [31]; the PMMA patterned substrate was firmly placed on the motorized translational stage as a lower substrate. Next, the upper blade (i.e., slide glass) was adjusted at 30 degrees, using a multi-axis-support right above the target substrate at a distance of ~100 µm. The prepared GO/AuNPs water solution (~50 μL) was injected into the restricted geometry (i.e., upper fixed blade and the patterned PMMA substrate), after which the capillary-held trapped GO/AuNPs meniscus could be formed. Subsequently, the translation stage traveled back and forth at a constant speed of 15 mm s^−1^ with an optimized deposition number (100 cycles) to coat the surface of the Si substrate between PMMA stripe patterns. Finally, selective removal of the PMMA patterns was performed to lift off the GO-AuNPs that are deposited in the undesired region (i.e., covered on top of the PMMA surface) using organic solvent. Then, the sample was dried by blowing N_2_ gas after thoroughly rinsed with deionized water and isopropyl alcohol (IPA). 

The patterned GO/AuNPs surface was then immobilized for 24 h with a drop of green fluorescent dye-labeled thiolated oligonucleotides deionized (DI) water solution (6-FAM-Q: 1-dimethoxytrityloxy-3-[O-(N-carboxy-(di-O-pivaloyl-fluorescein)-3-aminopropyl)]-propyl-2-O-succinoyl−longchain alkylamino-CPG; typically, 25 μL). In this process, the GO/AuNPs sample was sealed with a glass coverslip surrounded with polydimethylsiloxane (PDMS) gasket to prevent water evaporation. After the immobilization, the sample surface was extensively rinsed with DI water to wash away unbonded oligonucleotide. The thickness of oligonucleotide adsorbed on the sample surface was ~7–10 nm when measured by the atomic force microscope.

### 2.4. Synthesis of Monolayer Graphene

A Cu foil was prepared for a conventional thermal CVD process [32]. Firstly, the Cu foil was located inside the quartz reaction tube and annealed for 50 min under H_2_/Ar (20 sccm/50 sccm) flow from room temperature to 1050 °C at a constant pressure (100 mTorr). Then, the reaction gas mixture, CH_4_/H_2_/Ar (20/60/50 sccm), was introduced into the reaction tube for 20 min for the graphene growth. Finally, the quartz tube was cooled down at 3 °C s^−1^ until it reached room temperature. For the transfer process for the grown graphene on the Cu foil, PMMA solution (Mw = 401 kg mol^−1^, PDI = 1.3, polymer source) in toluene (10 wt%) was spin-coated onto the top of the graphene-grown Cu foil at 3000 rpm for 30 s. The Cu foil under the PMMA/graphene film was then fully etched in an optimized etchant (Etchant Type I, Transene). Finally, the graphene was transferred onto thermally grown SiO_2_ (300 nm, gate dielectric layer) on a heavily doped p-type silicon wafer (resistivity of ~0.001–0.005 Ω∙cm), followed by the dissolving of the PMMA layer with acetone.

### 2.5. Fabrication of Microfluidic Channel

The micro-patterned master mold for the microfluidic channels was initially fabricated through photolithography process using GXR 601 positive photoresist (AZ-GXR 601, AZ Electronic Materials, Luxembourg) on a Si substrate [33]; the patterned substrate was used as a positive mold to engrave PDMS microfluidic channels. The mixture of PDMS (Sylgard 184, Dow Corning, Midland, MI, USA) with prepolymer curing agent ratios of 10:1 was degassed for 30 min. PDMS prepolymer mixture was then poured onto the prepared patterned photoresist/Si wafer and cured for 2 h at 80 °C. By peeling off the cured PDMS from the master mold, a replicated microfluidic channel can be produced. To integrate the PDMS microfluidic chips on the arrays of graphene-based BioGFET, it requires a surface treatment step to generate strong and conformal bonds between interfacial surfaces. UV ozone treatment was performed on top of the PDMS that activates the surface of the PDMS by replacing Si–CH_3_ bonds with Si–OH groups, and then bonded with the BioGFET formed on SiO_2_/Si substrate (Figure S5) [34].

### 2.6. Graphene Surface Modification: Biotinylation and Streptavidin Detection

The graphene-based BioGFET substrate was sequentially modified with poly(ethylene imine) (PEI, Mw = 25 K, Sigma Aldrich), poly(ethylene glycol) (PEG, Mw = 10 K, Sigma Aldrich), and DI water solution at room temperature, using a drop-based immobilization or in microfluidic channels. After the PEI/PEG specific surface modification, the channel regions were exposed with a drop of 15 mM biotin-N-hydroxy-succinimide (NHS) ester (Sigma Aldrich) in dimethylformamide (DMF) solution, followed by incubation at room temperature for 12 h. Next, the surface-functionalized device was cleaned with DMF and DI water and dried by blowing N_2_ gas. Separately, streptavidin was dissolved in the 0.01 M buffer solution (phosphate-buffered saline, PBS, pH 7.2); the prepared protein solution was ready to be bound or conjugated on the biotinylated graphene channel surface of BioGFET in the microfluidic channel device for real-time detection.

### 2.7. Characterization and Measurements

The samples were observed by optical microscopy (Olympus BX51 and IX81-F72) in reflective mode, scanning electron microscopy (SEM, Carl Zeiss AG-SUPRA 40 VP, 5–10 kV), and transmission electron microscopy (TEM, TALOS F200X, operated at 200 kV). The surface of morphologies was collected using an atomic force microscopy (AFM) in non-contact mode (Park System XE-100). Raman spectra for GO and CVD grown graphene were obtained by Raman spectroscopy with 532 nm laser excitation (UniNanoTech, UniRam-II). In addition, after patterning the graphene channel using gradient stripe patterns of PMMA etch-mask with a condition of the mild oxygen plasma etching (100 W, 100 sccm, 60 s), the source/drain electrodes were prepared by standard photolithography (AZ-GXR 601) and lift-off process to define patterns of Cr (5 nm)/Au (20 nm) electrodes on patterned graphene/Si substrate. The channel lengths (*L*), defined as the distance between source and drain, and channel width (*W*) were fixed at 7 μm and 50 μm, respectively; the graphene-based BioFET measurement was carried out using a semiconductor parameter analyzer (Agilent 4156A) in ambient condition.

## 3. Results and Discussion

### 3.1. Evaporative Self-Assembly of PMMA Microstructures in Roll-to-Plate Geometry

Figure 1a,b presents a schematic illustration of the drying mediated self-assembly process to produce patterned PMMA stripe arrays, facilitating a moving roll on a flat substrate (i.e., SiO_2_/Si). In the experimental scheme, we designed a simple and viable route to spontaneously produce a set of highly ordered PMMA stripes. The first step involves the loading of a solution of PMMA in the restricted geometry, composed of an upper cylindrical roll on the lower receiving substrate placed on the stage. This approach is similar, but slightly different compared with our previous studies [35,36], which are the fixed confined geometry in a capillary-held solution and the surface tension driven instabilities of thickness gradient polymer thin films. Hence, PMMA polymer patterns (i.e., lines and spacing stripes) were created by the repetitive pinning/depinning cycles at the three-phase surface contact line when a meniscus of the polymer solution was forced to migrate by upper moving roll in the confined geometry, as described previously [34]. As the solvent evaporates, a constant laminar flow transports the polymer to the capillary edge from the bulk area of the trapped solution to the pinned contact line; it forms a well-known ‘‘coffee ring’’ effect (the left panel in Figure 1c) [37]. Once the meniscus is pinned, the edge of the contact line starts to shrink owing to the evaporation of the solvent and moving upper cylindrical roll. This combinative confined geometry controlled the gradual reduction of the contact angle of the meniscus that induces the continuous deposition of polymer chains. At this moment, the protruded ends of the PMMA polymer chains smoothly entangled onto the contact line deposited, and the meniscus slowly receded following the moving direction with micron scale (the middle panel in Figure 1c). Finally, the contact angle reaches a critical value, at which the depinning force (i.e., capillary force) becomes larger than the pinning force, forming a PMMA stripe patterned by this sequential event until the contact line pin at a new position (i.e., slip). Thus, the newly pinned contact line recovers the initial contact angle (right panel in Figure 1c). This repetitive cyclic stick-slip motion at the three-phase contact line results in successive PMMA patterns, as shown in Figure 1a. The use of the rational design of restricted geometry modulates the solvent evaporation of the meniscus and the motions of the contact lines, exhibiting a spontaneous formation of the textured PMMA surface with regular micron intervals (i.e., lines-spacing). Figure 1d shows a representative micrograph of PMMA stripe patterns formed on a SiO_2_/Si substrate in a large area on the *roll-on-plate* geometry (*C* = 0.125 μg mL^−1^). Notably, highly ordered structures were rapidly created by a one-step process, which showed extremely regular periodic stripes with a sharp contrast over the entire substrate without undulations locally up to ~200 nm at the final stage of the process (inset in Figure 1d). It is worth noting that the pattern dimensions and density of stripes depend on the speed of the moving meniscus and the evaporation rate of the solvent. However, in our case, we fixed the moving speed of the roll of 7 μm s^−1^ and the evaporation rate of toluene in a sealed chamber. Therefore, the concentration of the polymer solutions can be the only variable parameter to control the pattern width and intervals with the optimized control of the moving cylindrical surface.

### 3.2. Concentration Effect on the PMMA Pattern Formation

**Figure 2:**
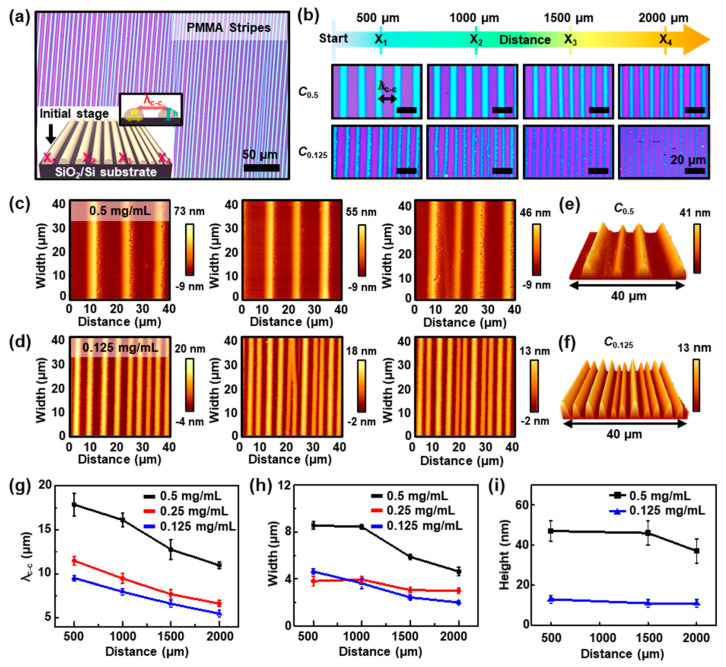
(**a**) An optical micrograph of a highly regulated array of PMMA stripes on the SiO_2_/Si substrate with a concentration of 0.125 mg mL^−1^; the inset is the schematic drawing, in which the value of λ_c-c_ indicates the typical center-to-center distance between two adjacent stripes; *X*_1_, *X*_2_, *X*_3_, and *X*_4_ denote the distances of PMMA stripes away from the initial stage. (**b**) Optical micrographs of periodically patterned stripe arrays of PMMA with varied concentrations (*C* = 0.5 and 0.125 mg mL^−1^); the images were measured at the different locations; scale bars are 20 μm for all images. (**c**,**d**) Atomic force microscopy (AFM) images of the PMMA stripes formed on the SiO_2_/Si substrate, collected from the initial (left) to final (right) stages from concentration with 0.5 mg mL^−1^ and 0.125 mg mL^−1^, respectively. (**e**,**f**) The 3D topological AFM images from the concentration of 0.5 and 0.125 mg mL^−1^, respectively; the scan position was in 2000 μm (*X*_4_ region) with a size of 40 × 40 μm^2^. (**g**–**i**) The measured gradient values of λ_c-c_, *w*, and *h* at different locations, depending on the concentration of the PMMA solution, respectively.

Figure 2 shows a collective set of the highly ordered PMMA stripes formed by cyclic stick-slip motions of the contact line at the different PMMA concentrations [38]. A magnified optical micrograph of PMMA patterns presents finely formed regular microstructures in a large area at the concentration of 0.125 mg mL^−1^, as shown in Figure 2a. As described earlier, the formation of periodic PMMA stripes was a direct consequence of controlled, repetitive ‘stick-slip’ motions of the contact lines and the competition of linear pinning force and nonlinear depinning force (i.e., capillary force) in the roll-on-flat geometry. Owing to the evaporating solvent, the nonvolatile solute (i.e., PMMA) can be condensed at the edge of the meniscus; the increase of the local viscosity of polymer solution caused polymer gelation in the process. After complete deposition of polymer in a certain period onto the SiO_2_/Si substrate, the pinning stress relaxed and the edge of the meniscus slowly replaced its position following the roll-moving direction like a receding tide for the next pinning. In addition, the stick-slip cycles mainly depend on the solution concentration in our system, as the pinning occurs when the polymer concentration reaches a certain critical value at the contact line. Reaching the threshold value repetitively, highly regular stripe patterns were spontaneously deposited under the fixed evaporation rate of the solvent (i.e., toluene). As shown in the graphical image in Figure 2a (inset), the dimension of the stripe-patterned PMMA structure on the substrate varies with the distance from the initial to the final stage; the center-to-center distance between adjacent stripes, λ_c-c_; width, *w*; and height of the pattern, *h*, gradually decrease, as denoted by *X*_1_, *X*_2_, *X*_3_, and *X*_4_ (i.e., the distances of PMMA patterns away from the initial stage). Figure 2b shows representative optical micrographs captured at each position. The periodicity of the gradient PMMA stripes can be defined by the concentration of polymer solution when the roll moves at a constant speed of 7 μm s^−1^. The images were collected from the 500 μm to 2000 μm in the order of *X*_1_, *X*_2_, *X*_3_, and *X*_4_, which extensively represents that extremely regular periodic stripe patterns, noted as λ_c-c_, and *w* were clearly decreased as the distance increased away from the early stage of the self-assembly process. This is mainly owing to the gradual change in the concentration of the polymer solution as the solvent evaporates between the roll and the substrate. In other words, more concentrated polymer solutions are more exhausted in the early stages of the process, so less consumable polymer solutes can be left at the final stage. In addition, the deposition time gradually decreases owing to the loss of the solvent volume during the roll-on-plate based deposition process [39].

To explore the surface morphology of the PMMA stripe patterns, we measured the surface using AFM. The topographical images of the highly-ordered PMMA patterns were obtained from the case of different solution concentrations, *C* = 0.5 and 0.125 mg mL^−1^. Figure 2c,d show AFM height images and the corresponding cross-sectional height profiles of the detailed morphological changes with changing of the concentration polymer solutions. The recession of λ_c-c_ and *h* was clearly observed; the height of the stripes at each of the high and low concentrations decreased progressively from *h* = 47 nm and 13 nm at *X*_1_ (left panel) to *h* = 46 nm and 11 nm at *X*_2_ (middle panel), and to *h* = 37 nm and 11 nm at *X*_4_ (right panel), respectively. In addition, Figure S1 shows the height profiles of the periodically patterned stripe arrays of PMMA obtained from different solution concentrations. It is noteworthy that this pattern formation process based on roll-on-flat geometry using a polymer solution was highly reproducible, appealing the unprecedented regularity. Intriguingly, some measured area macroscopically revealed perturbed surface features around the edge of stripes (right panel in Figure 2c), which may be attributed to the fast depletion of polymer solution during the slip process with less deposition duration for the polymer chains entanglement, thus slight dewetting occurred [40]. As presented in 3D AFM images (Figure 2e,f), the highly regular stripes appeared at the *X*_4_ region, which clearly revealed the sharp critical dimensions and spacing in nanometer height scale with different densities in the same scanned area (40 × 40 μm^2^), depending on the polymer concentration (i.e., *C* = 0.5 and 0.125 mg mL^−1^). When the concentration of the polymer solution increased in the process, more nonvolatile solute (i.e., PMMA) can be deposited at the capillary edge, leading to a wider width of the stripes because the “stick” period at the contact line becomes longer with the same contact angle recovery. Thus, an increased distance of the contact line to the next new position and a wider pattern interval can be yielded. According to the measured images, the gradient trend defined by λ_c-c_ and *w* of the PMMA stripes at the different concentrations (*C* = 0.5, 0.25, and 0.125 mg mL^−1^) was closely examined as a function of positions, *X*_n_ (n = 1 to 4). In the case of *C*_0.125_, critical dimensions were progressively changed from λ_c-c_ = 9.50 ± 0.33 μm and *w* = 4.63 ± 0.23 μm at *X*_1_ (500 μm), to λ_c-c_ = 7.98 ± 0.37 μm and *w* = 3.63 ± 0.42 μm at *X*_2_ (1000 μm), to λ_c-c_ = 6.61 ± 0.38 μm and *w* = 2.44 ± 0.23 μm at *X*_3_ (1500 μm), and to λ_c-c_ = 5.49 ± 0.39 μm and *w* = 2.00 ± 0.15 μm at the region *X*_4_ (2000 μm). Moreover, *h* values at the typical concentration were extracted by the series of AFM measurements as a function of *X* (Figure 2i), which indicated that *h* was slightly changed until it reached *X*_3_ in both cases (i.e., *C*_0.5_ and *C*_0.125_). Compared with the case of *C*_0.5_, the trapped solution possessed a lower amount of PMMA solute in the relatively dilute solution (i.e., *C*_0.125_) to create local stripe patterns by pinning–depinning of the contact lines; the duration of these events was much shorter. Therefore, the evaporative loss of toluene was also less under the constant guidance of the moving upper surface with continuous solvent evaporation, which caused the relatively uniform cyclic stick-slip motions, as indicated by the smaller *h* and *w*, as shown in Figure 2f. We believe that this optimized concentration regime is highly advantageous to manipulate highly uniform arrays of polymer stripe patterns in a scalable manner for useful applications such as template and etch masks [41].

### 3.3. Template-Assisted Formation of GO/AuNPs Stripe Patterns

GO is known to be characterized by atomically thin and 2D carbon nanomaterials that are embedded with carboxyl groups on the edges and hydroxyl and epoxy groups on the basal plane. The aromatic structural surface of GO makes it an ideal platform by functionalization for the adsorption of chemical moieties or biomolecules [42], while its high surface-to-volume ratio is known to influence sensing signals in bio-applications. Besides, metallic gold nanoparticles (AuNPs) have been considered the most stable of the noble metal NP class, which is especially characterized by the unique absorption and scattering properties dominated by the localized surface plasmon resonance (LSPR) [43]; particularly, the LSPR signal of AuNPs has been known to amplify fluorescence signals. Moreover, its resistance to surface oxidation and chemical inertness has attracted extensive use in many applications [44].

**Figure 3:**
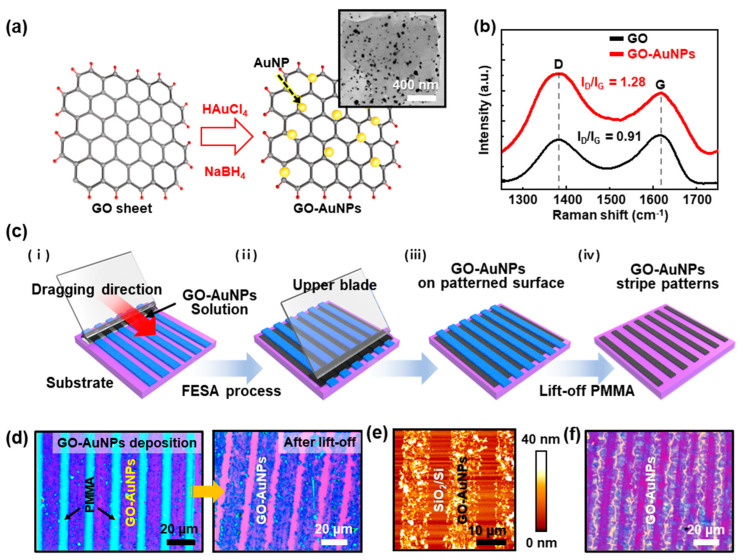
(**a**) Schematic drawing of the synthesis of graphene oxide (GO)/AuNPs and transmission electron microscopy (TEM) image (inset); AuNPs uniformly distributed along with the basal plane in GO sheets via co-reduction process. (**b**) Raman spectra of pristine GO and GO/AuNPs. (**c**) A series of schematic illustrations for the template-assisted self-assembly of stripe patterned GO/AuNPs surface. (**d**) Optical micrographs of the patterned film surface of GO-AuNPs on the SiO_2_/Si substrate after the lift-off of the stripe patterned PMMA from *C* = 0.125 mg mL^−1^; arrows in highly magnified images show initial sacrificial PMMA patterned arrays (left) and, after the lift-off process, left behind the stripe patterned GO/AuNPs (right). (**e**) A typical AFM height image of stripe patterned GO/AuNPs’ surface on the SiO_2_/Si. (**f**) Optical micrographs show stripe patterns of wrinkled surface structures.

To fully utilize the fabricated PMMA stripes, the template-assisted assembly of NP-modified GO nanosheets was performed as shown in Figure 3. As schematically represented in Figure 3a, the GO/AuNPs complex can be synthesized via a co-reduction process. A sheet of GO/AuNPs hybrid was examined by TEM (Figure 3a, inset), which demonstrated the relatively homogeneous distribution of AuNPs on the surface of GO sheets. The AuNPs might be preferentially anchored to active defect sites of GO (i.e., dangling bonds and oxygen vacancies). As previously reported [45], the GO surfaces are easily decorated with AuNPs because the exfoliated graphene oxide nanosheets are negatively charged owing to the hydrolysis and the well-known HAuCl_4_ precursors present at high pH owing to the hydrolysis of the [AuCl_4_]^−^. The electrostatic repulsion between the negatively charged exfoliated GO nanosheets and anionic Au precursors leads to the homogeneous GO-[AuCl_4_]^−^ suspension for a period, and then the affinity of Au^3+^ with the GO nanosheets owing to the formation of the grafted hydroxy-Au compound through the replacement reaction between HAuCl_4_ precursors and hydroxyl groups attached to the defects of GO nanosheets; the absorption of Au^3+^ to GO surface can be proceeded by Galvanic displacement, which induces the deposition of AuNPs on the GO sheets in the aqueous solution by the autocatalytic reduction. This reduction of GO-[AuCl_4_]^−^ could be promoted by adding the gallic acid (3,4,5-trihydroxy benzoic acid) as a reducing agent and stabilizer; thereby, the Au^3+^ can be partially reduced and the subsequent absorption on the exfoliated GO sheets is triggered simultaneously. Raman spectroscopic analysis was also performed to investigate the structural characteristics of GO and GO/AuNPs sheets, as shown in Figure 3b. The Raman spectrum of GO film displays two main peaks at ~1349 cm^−1^ (D band) and ~1600 cm^−1^ (G band), where the D band arises from the vibration of carbon atoms with dangling bonds in-plane terminations of disordered graphite, and the G band originates from the in-plane stretching of ordered sp^2^-bonded carbon atoms in the hexagonal lattice. In the case of the as-prepared GO/AuNPs sheets, the enhanced intensity and red-shift of Raman spectrum with the increased *I*_D_/*I*_G_ ratio of ~1.28 were observed, as compared with that of ~0.91 on the pristine GO sheets. These results indicate that the GO sheets were partially reduced by the combinatory reactions with the structural defects of GO sheets (e.g., vacancies or defective edges).

Figure 3c represents a schematic illustration of the template-assisted self-assembly process to create stripe patterns composed of GO/AuNPs composite nanomaterial. The prepared gradient polymer stripes on a SiO_2_/Si substrate were used as a sacrificial template to guide the formation of aligned arrays of GO/AuNPs stripes. When the aqueous GO/AuNPs solution (15 μL) was injected into the confined geometry of the upper blade on the PMMA stripe patterned substrate, the GO/AuNPs solution spread along with the different wetting in the restricted area, forming a meniscus by the capillary reaction (Figure 3c, (i)). The trapped GO/AuNPs solution was guided back-and-forth over the PMMA stripe patterned substrate using the motorized stage at a constant speed (15 μm s^−1^) with a preprogrammed number of cycles (~100), controlling the liquid thin layer of the GO/AuNPs solution. Specifically, the periodically alternating microstructure in hydrophobic PMMA stripes and hydrophilic SiO_2_ stripes (i.e., exposed areas) provided preferential wettabilities for the GO/AuNPs composites dispersed in DI water [46]. As the water solvent evaporated, the liquid thin films of GO-AuNPs naturally receded from the PMMA stripes, driven by the dewetting. The GO/AuNPs were then pushed to the SiO_2_/Si stripes underneath of the receding waterfront, following the evaporation-induced capillary flow (Figure 3c, (iii)). After the complete evaporation, GO/AuNPs stripes were achieved after selective removal of PMMA with organic solvent (i.e., lift-off). Figure 3d shows the representative optical micrographs of gradient stripes GO/AuNPs stripes over a large area utilizing the gradient templates of PMMA stripe patterns (Figure S3). The dimensional distributions of the GO/AuNPs stripes were highly complementary to those original PMMA stripes and were not disrupted during successive dissolving of PMMA with an organic solvent. To observe the surface characteristics of the templated GO/AuNPs stripes, AFM measurements were performed as shown in Figure 3e, in which the close examination clearly revealed the perfect pattern-transfer of the stacked GO/AuNPs sheets without the residues in between the adjacent stripes by the lift-off process. A magnified AFM image is presented in Figure S2; it revealed that the maximum height of stripes of the GO/AuNPs was ~20–40 nm at the center region, with gradual thickness variation. It is important to worth noting that the thickness of the templated GO/AuNPs correlated well with the values from the template of PMMA stripes at *X*_4_ (*C* = 0.5 mg mL^−1^). As shown in Figure S1, the nanoscale thickness of self-assembled PMMA stripes was a perfect fit to create an ultrathin layer of GO/AuNPs through templating the surface structures. This stacking strategy for the GO/AuNPs can be expanded by varying the concentration of the GO/AuNPs solutions; for example, stacking layers in stripe arrays were produced at the specific location (i.e., *X*_1_) as presented in Figure 3d. Moreover, interestingly, some wrinkle structures at the center regions are shown in Figure 3f. This was attributed to the larger evaporative loss of solvent at the edges of the PMMA templates compared with the interior of the evaporative GO/AuNPs solution when the GO/AuNPs sheets were pushed into the confined areas (i.e., SiO_2_ stripes) defined by the patterned PMMA templates during the evaporative assembly process [47].

### 3.4. Selective Immobilization of Fluorescein-Labeled DNAs on the Patterned GO/AuNPs

**Figure 4:**
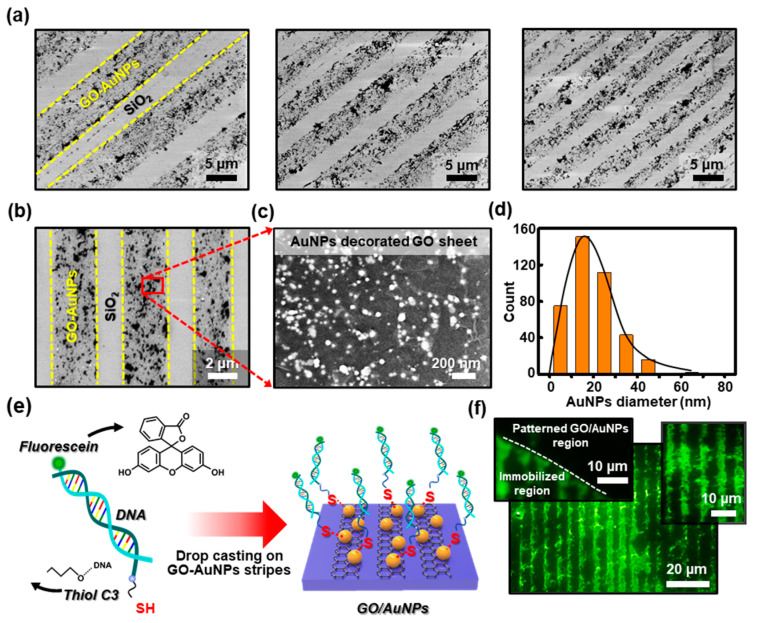
(**a**) A set of scanning electron microscopy (SEM) images collected from the different locations, presenting close-packed arrays of GO/AuNP stripes formed on a substrate in a gradient fashion. (**b**) A representative SEM image of the stripe patterned GO/AuNPs arrays configured with sharp contrast. (**c**) A highly magnified SEM image of densely embedded AuNPs in GO sheets, corresponding to the surface of the marked area shown in (**b**). (**d**) Histogram of the diameter of the AuNPs distributed on the stripe patterned GO sheet. (**e**) Schematic illustration of the DNA immobilization on the stripe patterned GO-AuNPs. (**f**) Fluorescent micrograph from the surface of the immobilized DNA arrays (inset: the white dotted line indicates the boundary between the DNA immobilized and non-patterned region (left), and the locally zoomed-in image (right)).

The immobilization technique of thiol-modified DNAs by self-assembly on the defined Au surface has been widely explored in the fabrication of DNA-based biosensors, specifically in the detection of DNA [48]. Our strategy to create patterned arrays of GO/AuNPs ultrathin films with unique structures can be readily combined with the advantageous properties of AuNPs to immobilize DNAs. By the simple reduction process, the embedded AuNPs in reduced graphene oxide (rGO) sheets may generate synergistic effects that can be expected to improve the performance of the DNA biosensors as an electrode surface. In this context, the patterned GO/AuNPs seem to be a simple strategy for linking biomolecules such as probe oligonucleotides, which allows an easy formation of layered ultrathin films with reasonable stability for extended chemical functionalization, allowing several reliable measurements [49]. To confirm the arrangement of AuNPs on the tethered surface, the samples were examined by the SEM after the complete preparation of the GO/AuNPs’ stripe patterns, as described in Figure 3d. Evidently, the representative SEM images at different locations appeared in Figure 4a, where the different densities of stripe micro-patterns in a gradient fashion were clearly observed in the same measured areas. A magnified image in Figure 4b, the stripes of GO/AuNPs sheets show a clear arrangement with up to ~2 μm intervals in a defined location (i.e., *C* = 0.125 mg mL^−1^ at *X*_4_). By zooming in on a specific area (Figure 4c), the measured surface indicates the characteristic features that are the formation of the densely packed AuNPs in the rGO sheets. Overall in the patterned structure, the reduced GO sheets showed stacked structures embedded with uniformly dispersed AuNPs. As presented in Figure 4d, the histogram shows the size distribution of AuNPs on the rGO sheet with the average particle size of ~21 nm determined from the SEM and TEM measurements. The buried AuNPs were not the perfect spherical shape, but exhibited a narrow deviation in the statistical size distribution of the measured surface area. In addition, the size of the AuNPs in the rGO sheets may attract considerable interest in the detection of fluorescently labeled thiol-modified DNA. The smaller the size of AuNP, the more the number of DNAs bound to one AuNP is limited owing to the repulsive force between the DNAs to be bound, so aggregation is expected to be appropriate. As schematically illustrated in Figure 4e, the fluorescently labeled thiol-modified DNA (i.e., 6-FAM-Q-labeled thiolated oligonucleotide) was functionalized on the rGO/AuNPs stripes to form a strong covalent bond on Au surfaces [50], producing a well-ordered structure. Generally, the thiol groups demonstrate the strong affinity towards the noble metal surfaces, allowing the formation of covalent bonds between the sulfur and Au atoms. By a simple immobilization process of DNA aqueous solution in a sealed PDMS microfluidic channel, a highly ordered patterned DNA surface was generated by self-assembly on the GO/AuNPs’ stripes. As shown in Figure 4f, the fluorescein-labeled DNA patterned arrays were selectively immobilized on the patterned GO/AuNPs in a large area; the emission of the green-emitting dye 6-FAM-Q is 520 nm. The insets in Figure 4f also show a direct comparison of the effectiveness of the stripe patterned GO/AuNPs’ surface selectively on the DNA immobilization. As a result, the patterned AuNPs’ buried rGO stripe patterns represent a more applicable capability as a strong candidate for AuNPs modified rGO-based electrodes in DNA biosensors. As the DNAs are a promising class of nanomaterial among others for constructing well-ordered micro/nanostructures for use in bio-related sensors and electronics, the self-assembly process presented here is advantageous mainly owing to the simplicity, low cost, and high stability in comparison with the other techniques. We believe that the biosensors for DNA detection based on the graphene nanomaterials such as GO or rGO might stimulate further research interest to expand the bio-sensing platform [51].

### 3.5. PMMA Stripes as Etch Masks for the Aligned Arrays of CVD Grown Graphene Sheets

**Figure 5:**
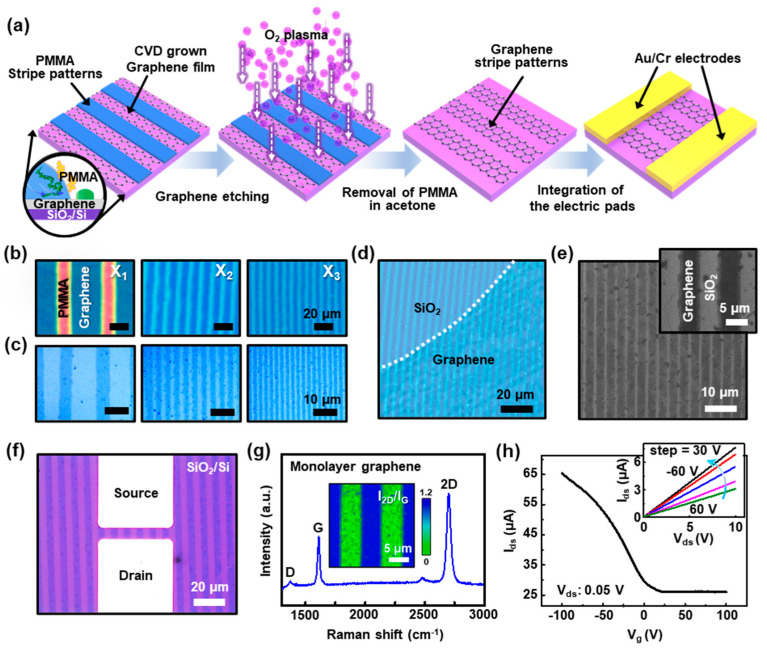
(**a**) Schematic description of a sequential process of patterning chemical vapor deposition (CVD) grown graphene using PMMA stripes as lithographic etch masks to build multichannel graphene field-effect transistors (GFETs). (**b**) A set of optical micrographs of the stripe patterned arrays of PMMA on graphene/SiO_2_/Si substrate; images were collected from the different regions (i.e., X_1_, X_2_, and X_3_). (**c**) A set of optical micrographs of micropatterned arrays of graphene films after the oxygen plasma and the subsequent removal of PMMA. (**d**) An optical micrograph of highly uniform PMMA stripe patterns formed on the graphene film; the white dotted line indicates the boundary between the graphene film and SiO_2_ surface. (**e**) SEM image of stripe patterned graphene arrays on the SiO_2_/Si substrate. (**f**) Typical optical micrograph of a back-gated multichannel GFETs, integrated with Au/Cr contact pads as the source and drain. (**g**) Raman spectrum measured from the graphene stripe on the SiO_2_/Si substrate, which indicates a monolayer in the graphene structure; the inset shows a Raman mapping image of the patterned graphene films. (**h**) Drain current (*I*_ds_) as a function of gate voltage (*V*_g_) for a source/drain bias (*V*_ds_) of 0.05 V; the inset shows an output characteristic of GFETs.

We further extended the present study to a smooth flat surface covered with CVD grown graphene toward the large scale etch-mask for the well-ordered graphene stripes by the self-assembly process based on the roll-on-plate geometry. The multichannel arrays of stripe patterned graphene being active in devices, formed with ultrathin patterned films, enables an improved current output, and may provide statistically beneficial device-to-device uniformity in properties even with the graphene films that are electronically heterogeneous [52]. Figure 5a illustrates a schematic of a sequential process to fabricate GFETs. The first step starts with the self-assembly of PMMA solution on a CVD grown graphene/SiO_2_/Si substrate by dragging the PMMA solution using roll-on-flat geometry, as described earlier. In the confined geometry, the drop of PMMA solution (typical *C* = 0.125 mg mL^−1^) was constrained, and the upper cylindrical roll was slowly moved at a constant speed of 7 μm s^−1^ to form highly aligned arrays of PMMA stripes (i.e., etch mask). As a result, the gradient PMMA stripe patterns successfully printed on the graphene surface after the complete assembly process (Figure 5b); different locations determine the variations in the density of the graphene stripes on the substrate, as observed at *X*_1_ to *X*_3_. Subsequently, a mild oxygen plasma process (100 W, 100 sccm, 60 s) was performed to etch away the exposed graphene films (i.e., uncovered areas by PMMA stripes). Figure 5c reveals the micropatterned arrays of graphene stripes with regular periodicity without the serious loss of resolution, perfectly matched to the original masking-patterns after the removal of the PMMA stripes using an organic solvent (i.e., acetone or toluene). Interestingly, Figure 5d indicates the uniform features of PMMA stripes, produced by the developed method, without the perturbation even at the heterogeneous boundary with the transferred graphene sheet (i.e., less than 1 nm) on the SiO_2_ surface; the white dotted line markedly indicates the boundary between the graphene and SiO_2_ region (uncovered by graphene). The aligned arrays of the stripe patterned graphene films were measured by SEM, as shown in Figure 5e, which confirms that the exposed graphene films were selectively removed and isolated as active multichannel arrays during the etching process using the stripe patterned PMMA etch mask. For GFET demonstration (i.e., back-gated FET), the prepared multichannel arrays of graphene films were integrated with the electric pads that were prepared by the conventional photolithography and lift-off process to define the source and drain by uniform thermal evaporation of Au (50 nm)/Cr (5 nm) electrodes on the patterned graphene/SiO_2_/Si substrate; the careful alignment of the mask on the patterned graphene surface was required. In the structure, the highly-doped Si with a SiO_2_ (300 nm) was underlying as a gate with a SiO_2_ gate dielectric layer. In some other cases, we transfer the graphene sheet onto the priorly prepared electric pads, and then the patterning process was performed using the confined geometry. Surprisingly, the fabrication of GFET proceeds with low levels of defects and local disorder, thereby leaving the patterned graphene supported directly on the probing Au electrodes (i.e., top-contact) on the SiO_2_/Si substrate, providing electrical isolation for each channel. To clarify the pattern formation of graphene on electrical pads on SiO_2_/Si substrate, the produced GFET was observed by an optical microscope, AFM, and SEM, as demonstrated in Figure S4.

Figure 5f shows a magnified optical micrograph of monolithically integrated GFET with multichannel (i.e., six active channels), providing a pair of electrical probing access points as the source/drain electrodes with the channel length and width of 7 and 50 μm, respectively. To investigate the quality of patterned graphene in the device structure, the surface of the samples was explored by Raman spectroscopy. As shown in Figure 5g, the Raman spectrum displays the characteristic 2D (~2697 cm^−1^), G (~1611 cm^−1^), and D (~1370 cm^−1^) peaks for monolayer graphene. In principle, the number of layers of graphene can be estimated by the ratio of 2D peak and G peak intensity to distinguish mono- and few-layer graphene. The intensity ratio of the G peak to the 2D peak (i.e., *I*_2D_/*I*_G_) resulted from the patterned monolayer graphene, where *I*_2D_/*I*_G_ was ~1.82, which also indicates the integrity of graphene was maintained regardless of the sequential patterning and etching process. Consequently, transfer characteristics of GFETs were measured by a semiconductor parameter analyzer under ambient conditions. Figure 5h presents a plot of the drain/source current (*I*_ds_) as a function of gate voltage (*V*_g_) at forwarding sweep between −100 and 100 V for a source/drain bias (*V*_ds_) of 0.05 V, and the characteristic output graph (V_g_: −60 to 60 from the top) is shown in the inset. The device exhibits Dirac points in the positive *V*_g_ region (~2.8 V) and represents the *p*-type transistor behavior, and the current modulation ratio (*I*_on_/*I*_off_) was ~2.5–3, which yielded the typical values from the micropatterned graphene FETs. This low level of on and off current ratio can be improved by the rational design of graphene (i.e., bandgap engineering) because the graphene bandgap can be open with lateral charge carrier confinement with suitable edge effects by narrowing the width of the graphene ribbons into nanoscale (i.e., graphene nanoribbons), as previously reported [53,54].

In addition, the linear-regime mobility, *μ*, can be calculated from the transfer curve, using the measured peak transconductance and typical model. We define the effective field-effect mobility of the FET device as

(1) $$\mu =∆\sigma /\left(C_{ox}∆V_{g}\right)=\left[\left(\frac{∆I_{ds}}{V_{ds}}\right)\left(\frac{L}{W}\right)\right]/\left(C_{ox}∆V_{g}\right)$$

where *L* and *W* are the channel length and width, respectively; *C_ox_* is the relative dielectric constant of the gate dielectric; and *I_ds_*, *V_ds_*, and *V_g_* are the drain-source currents, the drain-source voltage, and the gate voltage, respectively. Within the measured data set, resulting primarily from device-to-device variations in properties, the typical hole mobility ranged from ~270 to ~320 cm^2^ V^−1^ s^−1^ for the GFET devices. From this demonstration, PMMA etch mask utilized by the roll-on-flat geometry was a highly reliable method for device fabrication with its excellent ability by optimizing the graphene growth conditions and manipulation techniques, identifying the integrated GFET structures and property study [55].

### 3.6. Graphene-Based BioGFETs for Biotin-Streptavidin Real-time Detection

**Figure 6:**
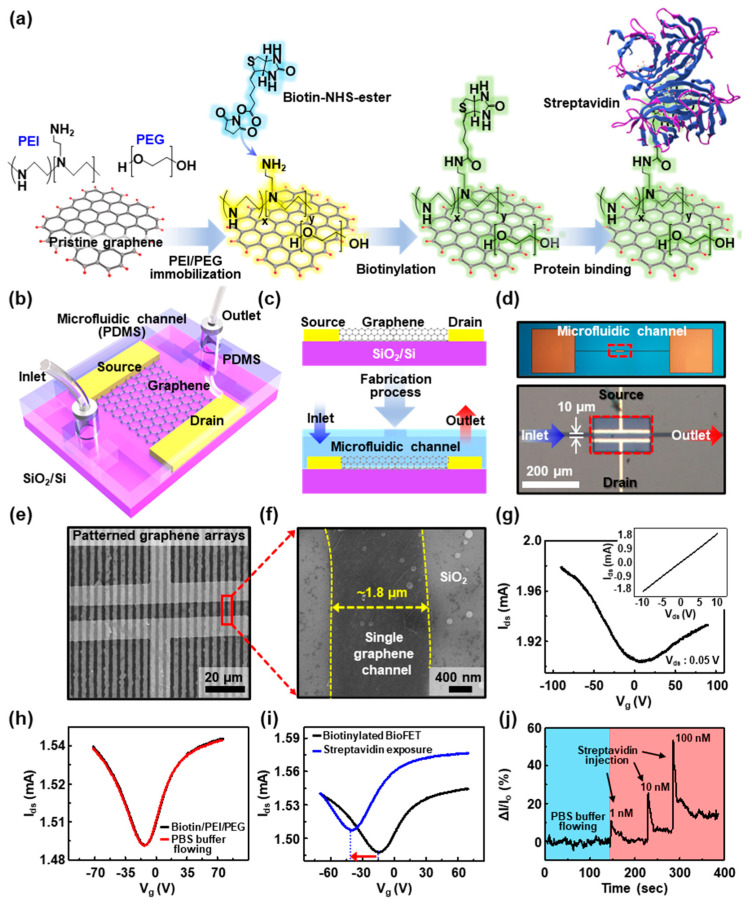
(**a**) Schematic diagram of the chemical modification steps on the graphene active channels to generate the BioGFET. (**b**) Structure of the BioGFET, integrated with a microfluidic channel. (**c**) A cross-sectional view of a microfluidic channel system built with BioGFET. (**d**) Optical micrographs of BioGFETs with a microfluidic channel. (**e**) SEM image of the chemically modified graphene multichannel, built with electric pads. (**f**) Zoom-in image of a single channel of the patterned graphene. (**g**) Gate voltage dependence of source-drain current before the surface modification with biotin/poly(ethylene imine) (PEI)/poly(ethylene glycol) (PEG); inset: current density–voltage (*I*–*V*) characteristics for a representative device. (**h**) Transfer characteristics according to the flowing of phosphate-buffered saline (PBS) buffer solution after biotin/PEI/PEG polymer coating on the graphene channels. (**i**) Transfer characteristics before and after streptavidin exposure on the active channels in BioGFET. (**j**) The signal was measured by monitoring the current as a function of time for different concentrations of streptavidin.

The prepared GFETs can be utilized as a biosensor platform (i.e., BioGFETs) to construct active functional channels because it is a zero bandgap semiconductor, and the bandgap can be tuned by surface modifications using biomolecules such as biotin terminal groups that can serve as a binding site for streptavidin [56,57]. Therefore, GFET as a promising transducer is beneficial in terms of the response for the charged molecules. Here, we present a simple approach to incorporate complement binding interactions on the graphene’s ultrathin body nature. Figure 6a describes the chemical modification steps on the micropatterned graphene channels to produce BioGFETs. In this experiment, the first step involved adopting the amine-rich aliphatic polymer, polyethyleneimine (PEI), to transform the characteristics of the pristine graphene surface. Subsequently, polyethylene glycol (PEG) was applied to prevent nonspecific binding onto the graphene surface, which was found to be most effective in resisting nonspecific adsorption of streptavidin. This PEGylated surface reduces the affinity of the graphene toward protein binding, mainly owing to its hydrophilicity, thus it has been used for reducing the binding of undesired species. Subsequently, sulfo-NHS-biotin was linked with the amine group of PEI/PEG/graphene channel surface; thereby, selective binding of streptavidin could be introduced by co-functionalization with PEI/PEG and biotin [58]. It is well known that there are four biotin-binding pockets in the streptavidin, but one or two of them can be bound to the immobilized biotin [58,59].

Figure 6b describes a basic structure of a BioGFET integrated with a microfluidic channel to detect streptavidin. The microchannel was composed of PDMS fabricated by photolithography, followed by a molding process to combine with the previously produced monolithically isolated multichannel GFETs connected with the external electrical probes. PDMS microfluidic channel was closed laminated with a precise alignment onto the surface of the device, preventing the leakage of the flowing or stored analyte. Cross-sectional views of the BioGFET with a microfluidic channel system are schematically shown in Figure 6c; the tubes were connected and sealed to the inlet and outlet of the microfluidic channel ends to delicately inject target streptavidin solution by the microsyringe pump. Figure 6d shows optical micrographs of as-prepared monolithically integrated GFET with a microchannel system operated with streptavidin-flow; the main reservoir was marked by a red dotted box in the lower image. In this microfluidic system, the flow field of fluids could be delicately sustainable by increasing the chance to rebind the analyte (i.e., streptavidin) at the chemically amenable interface (i.e., biotinylated graphene films) under the fluid streams by decreasing the length of diffusion. An example of BioGFET appears in Figure 6e,f, where aligned arrays of active components (i.e., stripe patterned graphene) with regular width (~1.8 μm) across the electrodes on the SiO_2_/Si substrate with areas that are comparable to the microfluidic dimensions of PDMS are observed. Thus, the design layouts represent both large sensing regions and ultrathin geometries in the structure for the biotin-streptavidin system, which is a standard model for protein–ligand interactions in our experiment scheme (Figure 6a) [60,61].

Figure 6g presents plots of the drain current (*I_ds_*) as a function of gate voltage (*V_g_*) with forward sweep for a source-drain bias (*V_ds_*) of 0.05 V for a typical BioGFET; the channel length, *L*, and the width, *W*, were 10 and 100 μm, respectively. The gate voltage of the neutrality point for the transfer curve was located at around 0 V, and the *p*-channel mode was dominant in the device operation based on the bottom-gated transistors with less effect of oxygen adsorption [62], estimated by the transconductance (*g*_m_) in *p*- and *n*-channel mode of ~1 µS and ~0.4 µS, respectively. The inset graph represents a typical *I*–*V* curve of a BioGFET and shows a solid linear fit with linear Ohmic behavior. After the sequential modification of biotin/PEI/PEG on the active channels in the microfluidic system, the transfer characteristics were apparently changed into ambipolar transport behavior. Complemental interaction of biotin occurs through covalent binding to the primary NH_2_ group, thereby promoting the overall electron-donating function of PEI/PEG and leading to a device characteristic that is consistent with the removal of electrons from the device, as shown in Figure 6h. Therefore, chemical functionalization of the graphene surface results in a doping effect, leading to an increased *n*-channel mode appearing that exhibits a slight shift of the Dirac point to the negative *V_g_* region. As the PEI molecules were involved in binding to biotin, the *p*-channel mode of conductance observed before the chemical modification was not fully recovered, and the negatively charged biomolecules, captured on the receptors, produce an electrostatic gating effect, which is transduced into an obvious current drop. Prior to use of the BioGFET for the streptavidin detection, a control experiment was performed to demonstrate the electrical response on the biotinylated polymer-coated device to 0.01 M PBS buffer flow through a PDMS microfluidic channel (the red curve in Figure 6h). It confirms no critical effect in the biotinylated graphene channels and represents that biotinylated polymer-coated BioGFET has excellent chemical stability and may yield high selectivity in the real-time monitoring system prepared as shown in Figure S5. For more information, Figure S6 illustrates the transfer characteristics of BioGFET by chemical modification of polymer coating (i.e., PEI/PEG immobilization) and biotinylation; in this case, we used a careful drop-casting right on top of the device channel. The device characteristic of pristine GFET (blue) shows the typical *p*-type characteristic owing to natural exposure to oxygen that can disrupt the π-conjugation of graphene and reduce electrical conductivity and carrier mobility. However, the ambipolar field-effect behavior was then observed from the sequential surface modification of PEI/PEG (black) and biotin/PEI/PEG on the GFET (red), respectively. Finally, the real-time electrical responses on the BioGFET to streptavidin were performed as shown in Figure 6i. The real-time electrical measurements were conducted at gate voltage range at a constant bias voltage of 0.05 V. Note that we stabilized the device with the tris-buffer solution before flowing the target molecule (i.e., streptavidin) to minimize the buffer solution effect into an electrical signal; any current change was not detected from the devices during buffer solution injection. The streptavidin dissolved buffer solution (167 nM in 0.01 M PBS) was then introduced into the PDMS channel using a microsyringe pump. The consequent streptavidin-biotin binding induced the complex doping effect on the graphene channels [63]; the presence of the charged target (i.e., streptavidin) binds a depletion of hole-carriers (i.e., an accumulation of electron carriers) in the graphene channels owing to the field effect. Such a doping effect causes a negative shift of Dirac point (i.e., −14 V to −40.5 V, indicated by an arrow) and the overall increase of the on-state in *n*-channel mode (i.e., increased by 0.04 mA), as observed in the graph. As extracted, the transconductances in the *p*- and *n*-channel were increased by ~6.7% (i.e., ~1.7 µS) and decreased by ~33% (i.e., ~2 µS), respectively; the *n*-channel mode of the transconductance shifted the notable magnitude in the operation, whereas the hole-conductance of the *p*-channel mode indicated small changes. This phenomenon might be attributed to the asymmetric doping effect in the graphene channels that was not exclusive to one dopant, but rather the result of more complicated transport mechanisms [56,63]. Figure 6j shows the partial changes measured in the current of a typical device as a function of time during exposure to solutions with various streptavidin concentrations. Here, electrons, generated by adsorbed streptavidin on the surface of biotin/PEI/PEG, move to the *n*-doped graphene channels. The current response was larger with higher concentrations, and the response tended to peak at a concentration of 100 nM. The increased carrier reacting to the concentration of streptavidin at the nanomolar concentration caused sensitive changes in the BioGFET in highly reproducible ranges. Conclusively, the operating behavior on the graphene channels of BioGFETs is primarily owing to the consequent *n*-type doping effect of the biotin complex, potentially upon the binding of streptavidin detection in the microfluidic channel. With reasonable sensitivity in biotinylated BioGFETs as a biosensor, the multichannel graphene arrays were effectively rendered and successfully demonstrated for a single protein monitoring with the help of the microfluidic channel system [64].

## 4. Conclusions

In summary, we have developed a simple, yet robust evaporative self-assembly strategy to spontaneously produce highly ordered PMMA stripes over a large area, facilitating a roll-on-plate geometry. This provides a unique environment to control the flow of PMMA solution by repetitive slip-stick motions with a limited evaporation rate between a moving cylindrical roll and a lower flat receiving substrate. The formation of stripe patterned arrays of PMMA was optimized by modulation of the solution concentration and the moving speed of the roll. To engineer the periodically patterned surface of PMMA stripes, they were served as an evaporative-guided template and lithographic etch masks as sacrificial patterned surfaces. Thus, highly ordered structural topographies and chemical features were beneficial to understand the relationship between the surface chemistries and biological systems. One example, the fluorescently labeled thiol-modified DNA, was immobilized on the arrays of GO/AuNPs by template-assisted self-assembly that may improve fluorescein behaviors by unique absorption and scattering properties for the biointerface. In addition, we demonstrated a lithographic approach to utilize the highly aligned arrays of PMMA stripes as etch masks for CVD grown graphene. With a simple surface modification process, such as co-functionalization with biotin/PEI/PEG on the conductive multichannel of graphene, molecular sensitive BioGFETs could be built to detect streptavidin with the help of the microfluidic channel system. Our presented study suggests that the stripe patterned arrays of polymer films as sacrificial templates can be a simple route to creating amenable biointerfaces and chemically patterned surfaces synergistically combined with the development of a new class of nanomaterials such as graphene and other 2D nanomaterials for a wide range of biosensor platform [65,66,67].

## Supplementary Materials

The following are available online at https://www.mdpi.com/2079-4991/10/8/1468/s1, Figure S1: The height profiles of the periodic PMMA stripes obtained from different concentration, C = 0.5 mg mL^−1^ (a) and 0.125 mg mL^−1^ (b). Figure S2: Close-up AFM images of GO-AuNPs stripe corresponding to the one portion of Figure 3e. The typical height of the GO-AuNPs stripe was ~30 nm. Figure S3: Patterned arrays of GO/AuNPs by the lift-off process of PMMA after the template-assisted deposition on the SiO_2_/Si substrate. (a) Optical micrographs after the GO/AuNPs’ deposition on the patterned PMMA stripes with different concentrations of 0.5, 0.25, and 0.125 mg mL^−1^. (b) After the removal of PMMA using an organic solvent. The scale bars are 10 μm. Figure S4: (a) Typical optical micrograph of micropatterned graphene channels on pair of Au/Cr electrodes, in which the back-gated multichannel GFET was built on the SiO_2_/Si substrate. (b) AFM image and height profile of micropatterned graphene, measured from the marked area in (a). (c) A magnified SEM image shows the graphene conformably integrated on the electric pads after the oxygen plasma and the removal of PMMA etch masks. Figure S5: (a) A photograph of the BioGFET integrated with a microfluidic system and the probing accesses. (b) A magnified view of the BioGFET as illustrated in Figure 6c. Figure S6: Transfer characteristics after the PEI/PEG polymer coating on the graphene channels (black), and transfer characteristics after the biotinylation on the active channels in BioGFET (red).

## References

[1] Birkholz O., Burns J.R., Richter C.P., Psathaki O.E., Howorka S., Piehler J. (2018). Multi-functional DNA nanostructures that puncture and remodel lipid membranes into hybrid materials. Nat. Commun..

[2] Liu Q., Wu C., Cai H., Hu N., Zhou J., Wang P. (2014). Cell-based biosensors and their application in biomedicine. Chem. Rev..

[3] Grazon C., Baer R.C., Kuzmanovic U., Nguyen T., Chen M., Zamani M., Chern M., Arquino P., Zhang X., Lecommandoux S. (2020). A progesterone biosensor derived from microbial screening. Nat. Commun..

[4] Li H., Liu X., Li L., Mu X., Genov R., Mason A.J. (2016). CMOS electrochemical instrumentation for biosensor microsystems: A review. Sensors.

[5] Panwar N., Soehartono A.M., Chan K.K., Zeng S., Xu G., Qu J., Coquet P., Yong K.T., Chen X. (2019). Nanocarbons for biology and medicine: Sensing, imaging, and drug delivery. Chem. Rev..

[6] Maduraiveeran G., Sasidharan M., Ganesan V. (2018). Electrochemical sensor and biosensor platforms based on advanced nanomaterials for biological and biomedical applications. Biosens. Bioelectron..

[7] Naveen M.H., Gurudatt N.G., Shim Y.-B. (2017). Applications of conducting polymer composites to electrochemical sensors: A review. Appl. Mater. Today.

[8] Anichini C., Czepa W., Pakulski D., Aliprandi A., Ciesielski A., Samorì P. (2018). Chemical sensing with 2D materials. Chem. Soc. Rev..

[9] Nekoueian K., Amiri M., Sillanpaa M., Marken F., Boukherroub R., Szunerits S. (2019). Carbon-based quantum particles: An electroanalytical and biomedical perspective. Chem. Soc. Rev..

[10] Tiwari J.N., Tiwari R.N., Kim K.S. (2012). Zero-dimensional, one-dimensional, two-dimensional and three-dimensional nanostructured materials for advanced electrochemical energy devices. Prog. Mater. Sci..

[11] Wang Y., Cai R., Chen C. (2019). The nano-bio interactions of nanomedicines: Understanding the biochemical driving forces and redox reactions. Acc. Chem. Res..

[12] Cai P.Q., Leow W.R., Wang X.Y., Wu Y.L., Chen X.D. (2017). Programmable nano-bio interfaces for functional biointegrated devices. Adv. Mater..

[13] Papageorgiou D.G., Kinloch I.A., Young R.J. (2017). Mechanical properties of graphene and graphene-based nanocomposites. Prog. Mater. Sci..

[14] Han T.H., Kim H., Kwon S.J., Lee T.W. (2017). Graphene-based flexible electronic devices. Mater. Sci. Eng. R Rep..

[15] Lv W., Li Z., Deng Y., Yang Q.H., Kang F. (2016). Graphene-based materials for electrochemical energy storage devices: Opportunities and challenges. Energy Storage Mater..

[16] Li X., Tao L., Chen Z., Fang H., Li X., Wang X., Xu J.B., Zhu H. (2017). Graphene and related two-dimensional materials: Structure-property relationships for electronics and optoelectronics. Appl. Phys. Rev..

[17] Bonaccorso F., Sun Z., Hasan T., Ferrari A.C. (2010). Graphene photonics and optoelectronics. Nat. Photonics.

[18] Hess L.H., Seifert M., Garrido J.A. (2013). Graphene transistors for bioelectronics. Proc. IEEE.

[19] Kim S.J., Cho H.R., Cho K.W., Qiao S., Rhim J.S., Soh M., Kim T., Choi M.K., Choi C., Park I. (2015). Multifunctional cell-culture platform for aligned cell sheet monitoring, transfer printing, and therapy. ACS Nano.

[20] Yang Y., Song Y., Bo X., Min J., Pak O.S., Zhu L., Wang M., Tu J., Kogan A., Zhang H. (2020). A Laser-engraved wearable sensor for sensitive detection of uric acid and tyrosine in sweat. Nat. Biotechnol..

[21] Kim J., Campbell A.S., de Avila B.E., Wang J. (2019). Wearable biosensors for healthcare monitoring. Nat. Biotechnol..

[22] Yang Y., Asiri A.M., Tang Z., Du D., Lin Y. (2013). Graphene based materials for biomedical applications. Mater. Today.

[23] Hong G., Diao S., Antaris A.L., Dai H. (2015). Carbon nanomaterials for biological imaging and nanomedicinal therapy. Chem. Rev..

[24] Justino C.I.L., Gomes A.R., Freitas A.C., Duarte A.C., Rocha-Santos T.A.P. (2017). Graphene based sensors and biosensors. Trends Anal. Chem..

[25] Fu W., Jiang L., van Geest E.P., Lima L.M.C., Schneider G.F. (2017). Sensing at the surface of graphene field-effect transistors. Adv. Mater..

[26] Kim J., Kim M., Lee M.S., Kim K., Ji S., Kim Y.-T., Park J., Na K., Bae K.-H., Kim H.K. (2017). Wearable smart sensor systems integrated on soft contact lenses for wireless ocular diagnostics. Nat. Commun..

[27] Hong S.W., Byun M., Lin Z. (2009). Robust self-assembly of highly ordered complex structures by controlled evaporation of confined microfluids. Angew. Chem..

[28] Khalil I., Yehye W.A., Julkapli N.M., Rahmati S., Sina A.A.I., Basirun W.J., Johan M.R. (2019). Graphene oxide and gold nanoparticle based dual platform with short DNA probe for the PCR free DNA biosensing using surface-enhanced raman scattering. Biosens. Bioelectron..

[29] Keighley S.D., Li P., Estrela P., Migliorato P. (2008). Optimization of DNA immobilization on gold electrodes for label-free detection by electrochemical impedance spectroscopy. Biosens. Bioelectron..

[30] Kim S., Han K.I., Lee I.G., Yoon Y., Park W.K., Hong S.W., Yang W.S., Hwang W.S. (2017). A zero-power, low-cost ultraviolet-C colorimetric sensor using a gallium oxide and reduced graphene oxide hybrid via photoelectrochemical reactions. Catalysts.

[31] Park R., Kim H., Lone S., Jeon S., Kwon Y.W., Shin B., Hong S.W. (2018). One-step laser patterned highly uniform reduced graphene oxide thin films for circuit-enabled tattoo and flexible humidity sensor application. Sensors.

[32] Li X., Colombo L., Ruoff R. (2016). Synthesis of graphene films on Cu foils by chemical vapor deposition. Adv. Mater..

[33] Jeon S., Kwon Y.W., Park J.Y., Hong S.W. (2019). Fluorescent detection of bovine serum albumin using surface imprinted films formed on PDMS microfluidic channels. J. Nanosci. Nanotechnol..

[34] Berdichevsky Y., Khandurina J., Guttman A., Lo Y.-H. (2004). UV/ozone modification of poly(dimethylsiloxane) microfluidic channels. Sens. Actuators B Chem..

[35] Bae D.G., Jeong J.-E., Kang S.H., Byun M., Han D.-W., Lin Z., Woo H.Y., Hong S.W. (2016). A nonconventional approach to patterned nanoarrays of DNA strands for template-assisted assembly of polyfluorene nanowire. Small.

[36] Li B., Jiang B., Han W., He M., Li X., Wang W., Hong S.W., Byun M., Lin S., Lin Z. (2017). Harnessing colloidal crack formation by flow-enabled self-assembly. Angew. Chem..

[37] Kang S.H., Shin Y.C., Hwang E.Y., Lee J.H., Kim C.-S., Lin Z., Hur S.H., Han D.-W., Hong S.W. (2019). Engineered “coffee-rings” of reduced graphene oxide as ultrathin contact guidance to enable patterning of living cells. Mater. Horiz..

[38] Hong S.W., Giri S., Lin V.S.Y., Lin Z. (2006). Simple route to gradient concentric metal and metal oxide rings. Chem. Mater..

[39] Hong S.W., Xia J., Byun M., Zou Q., Lin Z. (2007). Mesoscale patterns formed by evaporation of a polymer solution in the proximity of a sphere on a smooth substrate: Molecular weight and curvature effects. Macromolecules.

[40] Hong S.W., Xia J., Lin Z. (2007). Spontaneous formation of mesoscale polymer patterns in an evaporating bound solution. Adv. Mater..

[41] Chang C., Sakdinawat A. (2014). Ultra-high aspect ratio high-resolution nanofabrication for hard X-ray diffractive optics. Nat. Commun..

[42] Adegoke O., Pereira-Barros M.A., Zolotovskaya S., Abdolvand A., Daeid N.N. (2020). Aptamer-based cocaine assay using a nanohybrid composed of ZnS/Ag_2_Se quantum dots, graphene oxide and gold nanoparticles as a fluorescent probe. Microchim. Acta.

[43] Mei Z., Tang L. (2017). Surface-plasmon-coupled fluorescence enhancement based on ordered gold nanorod array biochip for ultrasensitive DNA analysis. Anal. Chem..

[44] Chen M.S., Goodman D.W. (2004). The structure of catalytically active gold on titania. Science.

[45] Jian J.M., Fu L., Ji J., Lin L., Guo X., Ren T.-L. (2018). Electrochemically reduced graphene oxide/gold nanoparticles composite modified screen-printed carbon electrode for effective electrocatalytic analysis of nitrite in foods. Sens. Actuators B Chem..

[46] Hong S.W., Jeong W., Ko H., Kessler M.R., Tsukruk V.V., Lin Z. (2008). Directed self-assembly of gradient concentric carbon nanotube rings. Adv. Func. Mat..

[47] Lee S.M., Song S.K., Yoon S., Chung D.S., Chang S.T. (2019). Liquid thin film dewetting-driven micropatterning of reduced graphene oxide electrodes for high performance OFETs. J. Mater. Chem. C.

[48] Peng H.P., Hu Y., Liu P., Deng Y.N., Wang P., Chen W., Ai-Lin L., Yuan-Zhong C., Xin-Hua L. (2015). Label-free electrochemical DNA biosensor for rapid detection of mutidrug resistance gene based on Au nanoparticles/toluidine blue-graphene oxide nanocomposites. Sens. Actuators B Chem..

[49] Liu F., Choi J.Y., Seo T.S. (2010). Graphene oxide arrays for detecting specific DNA hybridization by fluorescence resonance energy transfer. Biosens. Bioelectron..

[50] Sandström P., Boncheva M., Åkerman B. (2003). Nonspecific and thiol-specific binding of DNA to gold nanoparticles. Langmuir.

[51] Blair E.O., Corrigan D.K. (2019). A review of microfabricated electrochemical biosensors for DNA detection. Biosens. Bioelectron..

[52] Jeong S.-J., Jo S., Lee J., Yang K., Lee H., Lee C.-S., Park H., Park S. (2016). Self-aligned multichannel graphene nanoribbon transistor arrays fabricated at wafer scale. Nano Lett..

[53] Kang S.H., Hwang W.S., Lin Z., Kwon S.H., Hong S.W. (2015). A robust highly aligned DNA nanowire array-enabled lithography for graphene nanoribbon transistors. Nano Lett..

[54] Li X., Wang X., Zhang L., Lee S., Dai H. (2008). Chemically derived, ultrasmooth graphene nanoribbon semiconductors. Science.

[55] Yi D., Jeon S., Hong S.W. (2018). Selectively patterned regrowth of bilayer graphene for self-integrated electronics by sequential chemical vapor deposition. ACS Appl. Mater. Interfaces.

[56] Lowe B.M., Sun K., Zeimpekis I., Skylaris C.-K., Green N.G. (2017). Field-effect sensors-from PH sensing to biosensing: Sensitivity enhancement using streptavidin−biotin as a model system. Analyst.

[57] Jeong C.K., Kim I., Park K., Oh M.H., Paik H., Hwang G.T., No K., Nam Y.S., Lee K.J. (2013). Electrical biomolecule detection using nanopatterned silicon via block copolymer lithography. ACS Nano.

[58] Farmer D.B., Golizadeh-Mojarad R., Perebeinos V., Lin Y.M., Tulevski G.S., Tsang J.C., Avouris P. (2009). Chemical doping and electron-hole conduction asymmetry in graphene devices. Nano Lett..

[59] Sedlak S.M., Schendel L.C., Gaub H.E., Bernardi R.C. (2020). Streptavidin/biotin: Tethering geometry defines unbinding mechanics. Sci. Adv..

[60] Star A., Gabriel J.P., Bradley K., Grüner G. (2003). Electronic detection of specific protein binding using nanotube FET devices. Nano Lett..

[61] Kim J.E., No Y.H., Kim J.N., Shin Y.S., Kang W.T., Kim Y.R., Kim K.N., Kim Y.H., Yu W.J. (2017). Highly sensitive graphene biosensor by monomolecular self-assembly of receptors on graphene surface. Appl. Phys. Lett..

[62] Loh K.P., Bao Q., Ang P.K., Yang J. (2010). The chemistry of graphene. J. Mater. Chem..

[63] Zhou X., Moran-Mirabal J.M., Craighead H.G., McEuen P.L. (2007). Supported lipid bilayer/carbon nanotube hybrids. Nat. Nanotechnol..

[64] Xu S., Zhan J., Man B., Jiang S., Yue W., Gao S., Guo C., Liu H., Li Z., Wang J. (2017). Real-time reliable determination of binding kinetics of DNA hybridization using a multi-channel graphene biosensor. Nat. Commun..

[65] Ryu B., Nam H., Oh B.-R., Song Y., Chen P., Park Y., Wan W., Kurabayashi K., Liang X. (2017). Cyclewise operation of printed MoS_2_ transistor biosensors for rapid biomolecule quantification at femtomolar levels. ACS Sens..

[66] Fu W., Feng L., Panaitov G., Kireev D., Mayer D., Offenhäusser A., Krause H.J. (2017). Biosensing near the neutrality point of graphene. Sci. Adv..

[67] Tyagi D., Wang H., Huang W., Hu L., Tang Y., Guo Z., Ouyang Z., Zhang H. (2020). Recent advances in two-dimensional-materials-based sensing technology toward health and environmental monitoring application. Nanoscale.

