# Supplementary material for: Graphene Templated DNA Arrays and Biotin-Streptavidin Sensitive Bio-Transistors Patterned by Dynamic Self-Assembly of Polymeric Films Confined within a Roll-on-Plate Geometry
Source: Nanomaterials (Basel). 2020 Jul 27;10(8):1468. doi: 10.3390/nano10081468 (PMC7466610; doi:10.3390/nano10081468)
Supplement: Supplementary file 1 [file nanomaterials-10-01468-s001.pdf]

nanomaterials

Supplementary Materials

Graphene Templated DNA Arrays and Biotin-Streptavidin Sensitive Bio-Transistors Patterned by Dynamic Self-Assembly of Polymeric Films Confined within a Roll-on-Plate Geometry

Sangheon Jeon [1,†], Jihye Lee [1,†], Rowoon Park [1,†], Jeonghwa Jeong [1], Min Chan Shin [1], Seong Un Eom [2], Jinyoung Park [3,*], and Suck Won Hong [1,*]

[1] Department of Cogno-Mechatronics Engineering, Department of Optics and Mechatronics Engineering, College of Nanoscience and Nanotechnology, Pusan National University, Busan 46241, Korea; sangheon.jn@gmail.com (S.J.); jihyelee970@gmail.com (J.L.);rowoon.p153@gmail.com (R.P.); 2jeong.s.o@gmail.com (J.J.); mch3024@gmail.com (M.C.S.)
[2] Substrate & Material Laboratory, LG Innotek Co., Ltd., Gumi 39419, Korea; sueom89@gmail.com
[3] School of Applied Chemical Engineering, Kyungpook National University, Daegu 41566, Korea
* Correspondence: jinpark@knu.ac.kr (J.P.); swhong@pusan.ac.kr (S.W.H.)
† These authors contributed equally to this work.

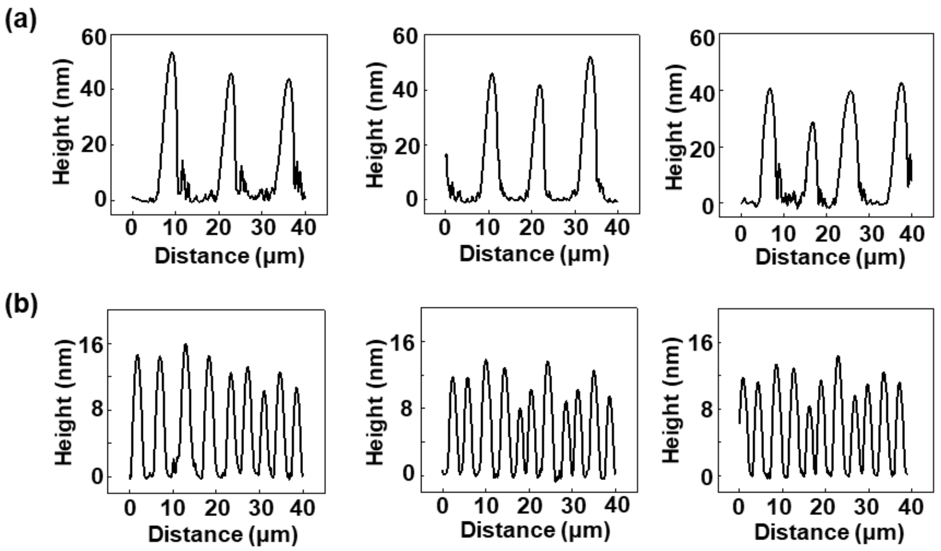

Figure S1. The height profiles of the periodic PMMA stripes obtained from different concentration, *C* = 0.5 mg mL^{-1} (**a**) and 0.125 mg mL^{-1} (**b**).

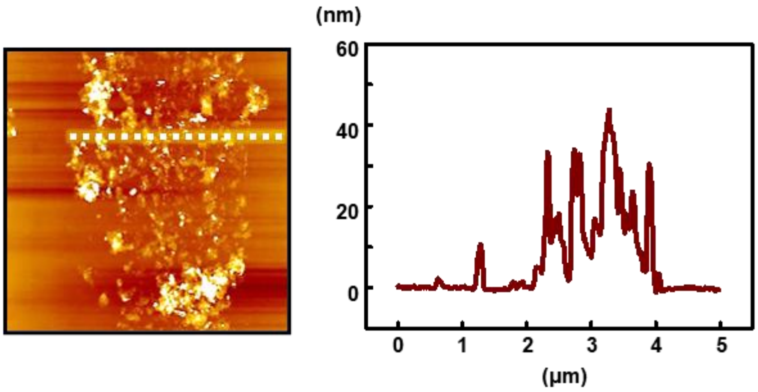

Figure S2. Close-up AFM images of GO-AuNPs stripe corresponding to the one portion of Figure 3e. The typical height of the GO-AuNPs stripe was ~30 nm.

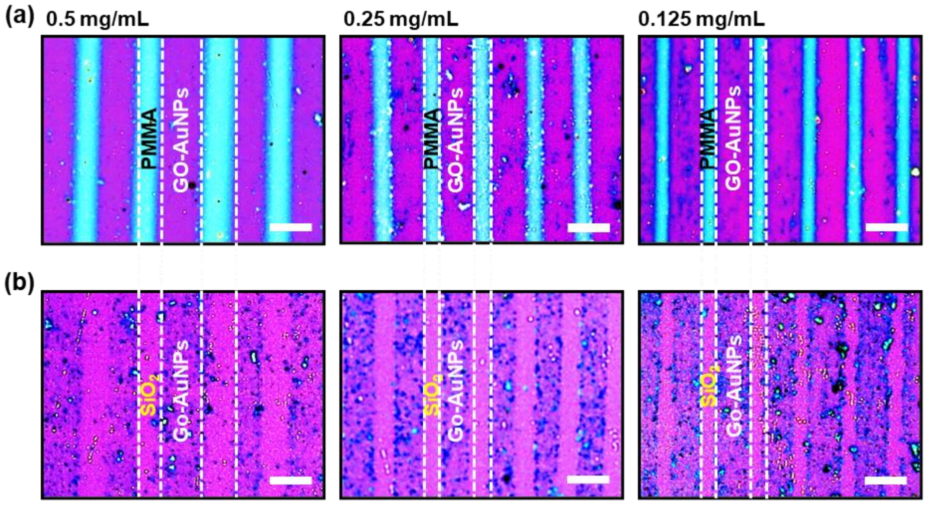

Figure S3. Patterned arrays of GO/AuNPs by lift-off process of PMMA after the template-assisted deposition on the SiO_2/Si substrate. (**a**) Optical micrographs after the GO/AuNPs deposition on the patterned PMMA stripes with different concentrations of 0.5, 0.25, and 0.125 mg mL^{-1}. (**b**) After the removal of PMMA using an organic solvent. The scale bars are 10 μm.

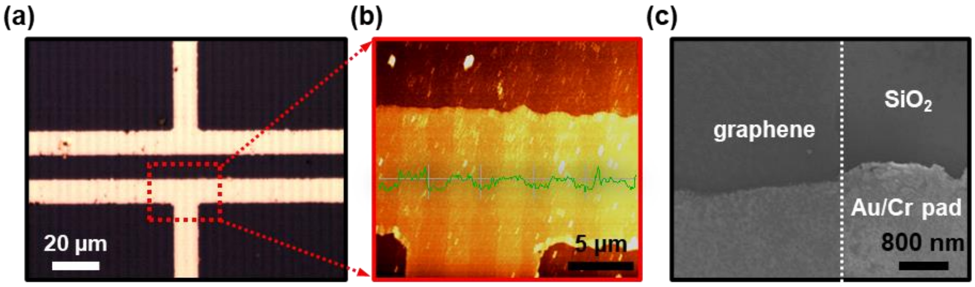

Figure S4. (**a**) Typical optical micrograph of micropatterned graphene channels on pair of Au/Cr electrodes, in which the back-gated multichannel GFET was built on the SiO_2/Si substrate. (**b**) AFM image and height profile of micropatterned graphene, measured from the marked area in (**a**). (**c**) A magnified SEM image shows the graphene conformably integrated on the electric pads after the oxygen plasma and the removal of PMMA etch masks.

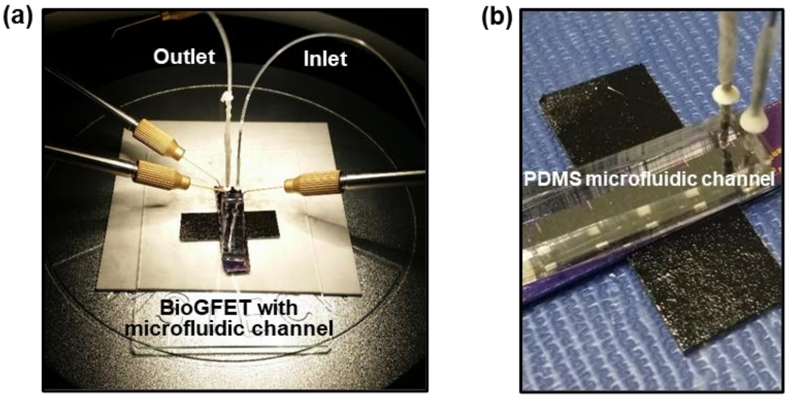

Figure S5. (**a**) A photograph of the BioGFET integrated with a microfluidic system and the probing accesses. (**b**) A magnified view of the BioGFET as illustrated in Figure 6c.

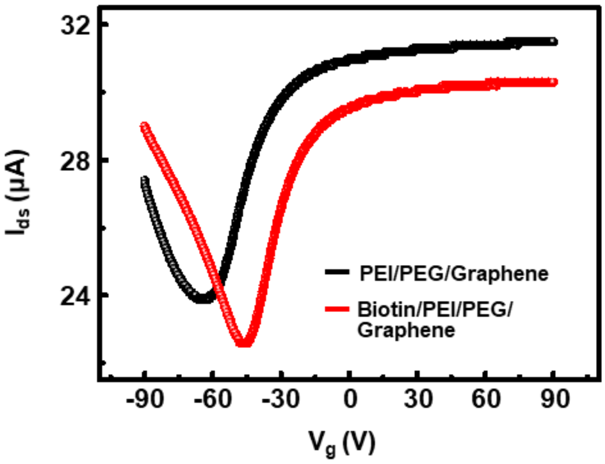

Figure S6. Transfer characteristics after the PEI/PEG polymer coating on the graphene channels (black), and transfer characteristics after the biotinylation on the active channels in BioGFET (red).
